# COVID-19 vaccine uptake among family caregivers of people with dementia: The role of attitudes toward vaccination, perceived social support and personality traits

**DOI:** 10.3389/fpsyg.2022.923316

**Published:** 2022-07-15

**Authors:** Francesco Bruno, Antonio Malvaso, Francesca Chiesi, Valentina Laganà, Rocco Servidio, Valeria Isella, Carlo Ferrarese, Federica Gottardi, Eloisa Stella, Federica Agosta, Massimo Filippi, Raffaele Di Lorenzo, Leslie R. Martin, Amalia Cecilia Bruni, Raffaele Maletta

**Affiliations:** ^1^Regional Neurogenetic Centre, Department of Primary Care, ASP Catanzaro, Lamezia Terme, Italy; ^2^Association for Neurogenetic Research, Lamezia Terme, Italy; ^3^Neurology Unit and Neurorehabilitation Unit and Neurophysiology Service, IRCCS San Raffaele Scientific Institute, Milan, Italy; ^4^Neuroimaging Research Unit, Division of Neuroscience, IRCCS San Raffaele Scientific Institute, Milan, Italy; ^5^Vita-Salute San Raffaele University, Milan, Italy; ^6^Section of Psychology, Department of Neuroscience, Psychology, Drug, and Child’s Health (NEUROFARBA), University of Florence, Florence, Italy; ^7^Department of Cultures, Education and Society, University of Calabria, Arcavacata di Rende, Cosenza, Italy; ^8^Department of Medicine and Surgery and Milan Center for Neuroscience (NeuroMi), University of Milano – Bicocca, Monza, Italy; ^9^I.P.S. Cardinal Gusmini Foundation ONLUS, Vertova, Italy; ^10^Novilunio Association, Padua, Italy; ^11^Department of Psychology, La Sierra University, Riverside, CA, United States

**Keywords:** family caregivers, dementia, COVID-19, vaccine hesitancy, vaccine uptake, personality traits, attitudes, perceived social support

## Abstract

People with dementia have an increased risk of contracting severe forms of COVID-19. Although in worldwide vaccination programs priority has been given to older people, having taken the vaccine does not totally eliminate the risk of contracting COVID-19 when one is in close contact with unvaccinated people. Thus, family caregivers’ choices to remain unvaccinated against COVID-19 could have potentially lethal consequences for their relatives. To our knowledge, this study represents the first attempt within the international literature to analyze COVID-19 vaccine uptake among family caregivers of people with dementia and to identify some of the psychological factors, related to COVID-19 and vaccination behavior, that could facilitate or hinder vaccine uptake. Contact information for family caregivers was obtained from five different centers and associations throughout the Italian territory. Data were collected from 179 respondents during July-September 2021 using a cross-sectional web-based survey design. More than 75% of the respondents indicated that had been vaccinated against COVID-19 and reported receiving vaccine information mainly from print or electronic newspapers (86%), followed by TV (81%) and families (64.2%). In multivariable logistic regression analyses, worries about unforeseen future effects was significantly related to COVID-19 vaccine uptake, indicating that family caregivers concerned about potential side effects of vaccines were less likely to have been vaccinated against COVID-19 (OR = 0.60, CI = 0.40-0.89). Openness to experience was also related to COVID-19 vaccine uptake, with family caregivers higher on this trait being less likely to have been vaccinated against COVID-19 (OR = 0.83, CI = 0.71-0.98). Implications for targeting of vaccine-related messages are discussed.

## Introduction

The Coronavirus Disease 2019 (COVID-19) pandemic has impacted both physical (Ng et al., 2020) and mental health (Vindegaard and Benros, 2020), particularly for vulnerable groups, including people with dementia (Cagnin et al., 2020). A recent meta-analysis demonstrated that dementia is associated with an increased risk of mortality due to SARS-CoV-2 infection (Hariyanto et al., 2020) for two key reasons. First, most people with dementia are elderly and have comorbidities that increase the severity of COVID-19’s clinical manifestations (Parohan et al., 2020). Second, the ApoE∈4∈4 genotype, often associated with dementia, significantly increases the likelihood of severe COVID-19 infections, as well as mortality (Kuo et al., 2020). These vulnerabilities make it especially important to minimize the exposure of people with dementia to the SARS-CoV-2 virus (Hariyanto et al., 2020). Vaccinations represent the most effective way to reduce the rate of many infections and the best hope for eradicating infectious diseases (Hajj Hussein et al., 2015; Fisher et al., 2020). However, the need for mass vaccination to control COVID-19 infections comes at a time of growing skepticism about vaccinations and a reluctance or refusal to vaccinate, despite the availability of the vaccine, especially in developed countries (World Health Organization, 2020; Lunz Trujillo et al., 2021). Several studies have reported a high rate of COVID-19 vaccine hesitancy both in the general population (Sallam, 2021) and in high-risk groups, such as cancer patients (Mejri et al., 2022; Servidio et al., 2022). Consequently, vaccine skepticism threatens COVID-19 immunization efforts (Ball, 2020; Taylor et al., 2020). Although the Italian COVID-19 vaccination program has given priority to high-risk groups, such as older people (Ministero della Salute, 2021), being vaccinated does not completely eliminate the risk of contracting COVID-19 when one is in close contact with unvaccinated people (Braeye et al., 2021), and especially when that contact is sustained (Ng et al., 2021), such as with family caregivers in a household.

Given the increased risk of contracting severe forms of COVID-19 and the consequently higher mortality for people with dementia (Hariyanto et al., 2020; Kuo et al., 2020; Parohan et al., 2020), family caregivers’ refusal to be vaccinated against COVID-19 could have potentially lethal consequences for their relatives. This makes understanding which factors best predict vaccination decisions in this group vitally important. Demographic characteristics (Sherman et al., 2021), attitudes toward vaccination (Breslin et al., 2021; Shacham et al., 2021), perceived social support (Moscardino et al., 2022), personality traits (Webster et al., 2022), fear of COVID-19 (Willis et al., 2021), understanding of COVID-19 (Ruiz and Bell, 2021), trust in health authorities (Petersen et al., 2021) and previous vaccination history for the seasonal influenza vaccine (Caserotti et al., 2021), have each been shown, in some way, to influence vaccine acceptance/hesitancy. Yet it is also recognized that these factors may be differently relevant for family caregivers because they are likely to be actively considering both themselves and the person for whom they provide care. Layered on top of this is the perceived efficacy of the vaccine for their elder dependent. Prior work has shown relatively low beliefs in the therapeutic benefit of drugs to treat dementia (Franchi et al., 2013) and it is possible that these low expectations may foster somewhat lower expectations for the health outcomes of dementia patients more generally. Therefore, the aims of this study were to: (i) examine the sources of information about COVID-19 vaccines for family caregivers of people with dementia, as well as their choices to receive COVID-19 vaccines; (ii) identify some of the psychological factors (i.e., attitudes toward vaccination, personality traits, social support) and other factors (i.e., understanding of COVID-19, fear of COVID-19, trust in health authorities, previous vaccination history for the seasonal influenza vaccine) relevant to COVID-19 vaccine uptake and to determine which are the best predictors of becoming vaccinated.

## Methods

### Participants and Procedure

Data were obtained from 179 family caregivers of patients with dementia. The response rate was 45%. Contact information on family caregivers was obtained from five different centers and associations (Regional Neurogenetic Centre, Lamezia Terme, CZ; I.P.S. Cardinal Gusmini Foundation ONLUS, Vertova, BG; Milan Center for Neuroscience - NeuroMi, Milano; Association for Neurogenetic Research, Lamezia Terme, CZ; Novilunio Association, Padua). A cross-sectional web-based survey design was adopted to limit face-to-face contacts due to the COVID-19 pandemic, using the free software Google Forms^®^. The online survey was distributed between July and September of 2021. An informational letter about the purpose of the study was mailed to all participants, along with a link to the questionnaires. Individuals were informed that participation in the study was voluntary, the survey was anonymous, and they could withdraw from the study at any time.

### Measures

#### Vaccination attitudes examination scale

Attitudes toward vaccination were evaluated using the Italian version of the Vaccination Attitudes Examination Scale (VAX-I scale; Martin and Petrie, 2017; Bruno et al., 2022). It consists of 12 items which can be divided into four subscales (mistrust of vaccine benefit, worries about unforeseen future effects, concerns about commercial profiteering, and preference for natural immunity), each indicated by three items. Each item is presented in the form of a statement, with responses on a 6-point Likert-type scale ranging from 1 (strongly disagree) to 6 (strongly agree). Higher scores on each subscale reflect stronger anti-vaccination attitudes. In our sample, internal consistency was good; Cronbach’s was, α = 0.85 for mistrust of vaccine benefit, α = 0.83 for concerns about commercial profiteering, α = 0.79 for preference for natural immunity and α = 0.81 for worries about unforeseen future effects.

#### Multidimensional scale of perceived social support

Perceived social support was evaluated using the Multidimensional Scale of Perceived Social Support (MSPP) (Zimet et al., 1988; Di Fabio and Busoni, 2008). The scale is composed of 12 items with response options on a 7-point Likert-type scale, ranging from 1 (absolutely false) to 7 (absolutely true). The instrument measures perceived social support from family, friends, and significant others. In our sample, Cronbach’s alpha reliabilities indicated excellent internal consistency, with α = 0.97 for family, α = 0.96 for friends, and α = 0.96 for significant others.

#### Ten-item personality inventory

Personality traits were measured using the Ten-Item Personality Inventory (I-TIPI; Gosling et al., 2003; Chiorri et al., 2015). This brief questionnaire assesses the Big Five personality dimensions: extraversion (E), agreeableness (A), conscientiousness (C), neuroticism (N), and openness to experience (O). Each of the ten items is rated on a 7-point scale ranging from 1 (strongly disagree) to 7 (strongly agree). Internal consistency in this sample was adequate, with Cronbach’s α = 0.87 for extraversion, α = 0.70 for agreeableness, α = 0.75 for conscientiousness, α = 0.81 for neuroticism, and α = 0.65 for openness to experience.

#### Fear of COVID-19 scale

The Fear of COVID-19 Scale (FCV-19S), designed by Ahorsu et al. (2020) and adapted to the Italian language by Soraci et al. (2020), was used to assess fear of COVID-19. The scale consists of 7 items rated on a 5-point Likert scale from 1 (strongly disagree) to 5 (strongly agree). In our sample, Cronbach’s alpha was good at α = 0.89.

#### Understanding of COVID-19

Understanding of COVID-19 was assessed using the scale proposed by Prasetyo et al. (2020). It consists of 5 items with responses on a 5-point Likert-type scale ranging from 1 (strongly disagree) to 5 (strongly agree). The scale showed good internal consistency, with α = 0.94.

#### Trust in health authorities

Trust in health authorities was measured using four items adapted from Caso et al. (2019). Each item (e.g., “The COVID-19 vaccination program is safe because it is approved by the Health Ministry”) was rated on a 5-point Likert-type scale ranging from 1 (strongly disagree) to 5 (strongly agree). Items were averaged to create the composite, with higher scores indicating greater trust. The scale showed excellent internal consistency, with α = 0.94.

#### Previous vaccination history for the seasonal influenza vaccine

Respondents were asked whether they had received any seasonal influenza vaccine in the past five years (yes/no).

#### COVID-19 vaccine uptake

Vaccine uptake was assessed by asking participants two questions: whether they had and whether their family member affected by dementia had received a vaccination against COVID-19 (yes/no).

#### Socio-demographics factors

Questions about sociodemographic characteristics were asked to family caregivers at the end of the survey. Because demographic correlates of COVID-19 vaccine uptake have been previously evaluated, we included them here and also gathered information on the demographic characteristics of the person receiving care in order to characterize the individual being cared for. Specifically, participants reported the gender, age, educational level, marital status, employment status, and economic condition for the family member with dementia, as well as themselves. They reported on exposure to COVID-19 for themselves and their family member (i.e., “have you been diagnosed with COVID-19?”; “has your family member affected by dementia been diagnosed with COVID-19?”). Family caregivers also reported clinical features of the experience for their family member (i.e., type of diagnosis, year of diagnosis, and current disease stage); indicated whether, before the COVID-19 pandemic, their family member left the house and/or attended dedicated services; and whether. by means of vaccines, they expected their family member would be able to re-establish the social habits that they had before. Finally, participants indicated their sources of information about the COVID-19 vaccine (i.e., print or electronic newspapers, TV, families, friends, personal doctor, radio, social networks, websites, or other sources). Response options for all these questions are presented in Tables 1-3.

**TABLE 1:** Demographic characteristics of family caregivers.

Variable	Categories	Frequency	Percentage
*Age*	29–82 years (Mean = 56.4; SD = 12.4)	−	−
*Gender*	Males Females	53 126	29.6 70.4
*Marital status*	Single In a relationship	33 146	18.4 81.6
*Education*	Less than high school High school Graduate Postgraduate (Masters, Ph.D. etc.)	8 100 57 14	4.5 55.9 31.8 7.8
*Occupation*	Employed Unemployed	97 82	54.2 45.8
*Economic conditions*	Extremely problematic Some problems Standard conditions Medium-high	4 40 98 37	2.2 22.3 54.7 20.7
*Contracted COVID-19 infection* *COVID-19 Vaccine uptake*	Yes No Yes No	20 159 135 44	11.2 88.8 75.4 24.6

**TABLE 2:** Demographics and clinical characteristics of patients.

Variable	Categories	Frequency	Percentage
*Age*	48–93 years (Mean = 76.3; SD = 9.9)	−	−
*Gender*	Males Females	54 125	30.9 69.8
*Marital status*	Single In a relationship	69 110	38.5 61.5
*Education*	Less than high school High school Graduate Postgraduate (Masters, Ph.D. etc.)	85 82 2 10	47.5 45.8 1.1 5.6
*Diagnosis*	Alzheimer’s Disease (AD) Frontotemporal Dementia (FTD) Dementia with Lewy bodies (DLB) Vascular Dementia (VD) Mixed (AD + VD)	110 26 11 25 6	61.5 14.5 6.1 14 3.4
*Disease’s stage* *Years from diagnosis*	Low grade Moderate Severe 2007-2021 (Mean = 2016 SD = 3.2)	32 92 55 −	17.9 51.4 30.7 −
*Contracted COVID-19 infection* *COVID-19 vaccine uptake*	Yes No Yes No	18 161 146 33	10.1 89.9 81.6 18.4

**TABLE 3:** Response rates of family caregivers to questions related to COVID-19.

Variable	Categories	n (%)	n total
*Did your family member with dementia leave the house before the COVID-19 pandemic began?*	Yes No	165 (92.2) 14 (7.8)	179
*Before the COVID-19 pandemic, did your family member with dementia attend dedicated services? (e.g., Day care center, Alzheimer’s cafe, etc.)*	Yes No	109 (65.3) 58 (34.7)	167
*Do you think that, thanks to the COVID-19 vaccine, your relative with dementia will be able to re-establish the habits they had before?*	Yes No	71 (48) 77 (52)	148
*Please, indicate where you get information on COVID-19 vaccines from (maximum 3 choices).*	Print or electronic newspapers TV Families Friends Confidential doctor Radio Social Networks Websites Other sources	155 (86.5) 145 (81) 115 (64.2) 113 (63.1) 105 (58.6) 97 (54.2) 85 (47.5) 70 (39.1) 69 (38.6)	179

### Statistical analyses

Data were analyzed in IBM SPSS Version 25 (IBM Corp., Released 2017). To explore and identify the factors related to COVID-19 vaccine uptake, correlational analyses followed by a multivariable logistic regression analysis were used, with odds ratios (ORs) and 95% confidence intervals (CIs) generated. “COVID-19 Vaccine Uptake” was entered as the outcome variable and predictors were selected *a priori*, based on their correlations with the criterion (i.e., marital status: single *vs.* in a relationship, worries about unforeseen future effects, concerns about commercial profiteering, preference for natural immunity, openness to experiences, and perceived social support received by family and significant others).

#### Sample size estimation

For logistic regression analyses, sample size is typically expressed in terms of events per variable (EPV), defined by the ratio of the number of events, (i.e., number of observations in the smaller of the two outcome groups) to the number of degrees of freedom (parameters) required to represent the predictors considered in developing the prediction model. Following Austin and Steyerberg (2017) recommending an EPV of 20, and Bujang et al. (2018) suggesting the rule of thumb *n* = 100 + EPV*i* (where *i* refers to number of independent variables in the final model), we estimated a target sample size of 200, assuming5 predictors in our model [100 + 20(5) = 200].

## Results

Demographics for family caregivers and patients are presented in Tables 1, 2, respectively.

Descriptive statistics are reported in Table 3. Most of the family caregivers declared that before the pandemic their family member with dementia left the house (92.2%) and attended dedicated services (65.3%). In addition, 48% of family caregivers stated that, by means of vaccines, their family members would be able to re-establish the social habits that they had before. Regarding the sources of information, we found that family caregivers received vaccine information mainly from print or electronic newspapers (86.59%), followed by TV (81%) and families (64.2%).

It’s evident from Table 4 that COVID-19 vaccine uptake for family caregivers was significantly associated with marital status, *r* = −0.17, *p* < 0.05 - with partnered family caregivers less likely to be vaccinated, worries about unforeseen future effects, *r* = −0.23, *p* < 0.01, Concerns about commercial profiteering, *r* = −0.15, *p* < 0.05, and preference for natural immunity, *r* = −0.16, *p* < 0.05. However, mistrust of vaccine benefit, *r* = 0.05, *p* > 0.05 was not significantly correlated with COVID-19 vaccine uptake. In terms of personality correlates, only openness to experience, *r* = −0.15, *p* < 0.01 was associated with vaccine uptake - those scoring higher on this dimension were less likely to have obtained the COVID-19 vaccine. Additionally, social support from family, *r* = −0.27, *p* < 0.001, and significant others, *r* = −0.17, *p* < 0.05 showed negative and significant correlations with COVID-19 vaccine uptake, indicating that those with perceptions of stronger support from family and significant others were less likely to be COVID-19 vaccinated, although perceived support from friends was unrelated.

**TABLE 4:** Correlation matrix with all potential predictor variables.

Variable	COVID-19 Vaccine Uptake
Family caregivers’ age	0.06
Family caregivers’ gender	0.04
Family caregivers’ marital status	−0.17*
Family caregivers’ education	0.08
Family caregivers’ occupation	–0.06
Family caregivers’ economic conditions	0.08
Worries about unforeseen future effects	−0.23**
Concerns about commercial profiteering	−0.15*
Preference for natural immunity	−0.16*
Mistrust of vaccine benefit	0.05
Openness to experience	−0.15*
Conscientiousness	–0.02
Agreeableness	0.05
Neuroticism	0.03
Extraversion	0.10
Perceived social support - family	−0.27***
Perceived social support - friends	–0.11
Perceived social support - significant others	−0.17*
Understanding of COVID-19	0.09
Trust in Health Authorities	–0.12
Fear of COVID-19	0.07
Previous vaccination history for the seasonal influenza vaccine	0.04

*Significant at 0.05 level. **Significant at 0.01 level. ***Significant at level 0.001. Point biserial correlations were used for correlations between one continuous and one dichotomous variable; phi coefficients were used for associations between two dichotomous variables. Spearman’s rho was used for correlation between one ordinal and one dichotomous variable.

The specific weight of each predictor is reported in Table 5. Worries about unforeseen future effects demonstrated a significant relation to COVID-19 vaccine uptake, indicating that family caregivers concerned about potential side effects of vaccines were less likely to have been vaccinated against COVID-19, OR = 0.60, 95% CI = (0.40, 0.89). Openness to experience also was related to COVID-19 vaccine uptake, with caregivers higher on this trait being less likely to have been vaccinated against COVID-19 (OR = 0.83, 95% CI = 0.71, 0.98). Marital status, concerns about commercial profiteering, preference for natural immunity and perceived social support received by family and significant others were not significant predictors of vaccination status.

**TABLE 5:** Multivariable logistic regression analysis with COVID-19 vaccine uptake as outcome variable.

Variable	β	SE β	Standardized^+^	Odds Ratio	Z	Wald Statistic	df	p	95% CI
*Marital Status*	−1.10	0.68	−0.43	0.33	−1.61	2.59	1	0.11	0. 09-1.27
*Concerns about commercial profiteering*	−0.05	0.17	−0.07	0.96	−0.28	0.08	1	0.78	0.69-1.32
*Preference for natural immunity*	−0.11	0.18	−0.14	0.90	−0.60	0.36	1	0.55	0.64-1.28
*Worries about unforeseen future effects*	−0.52	0.20	−0.64	0.60	−2.52	6.33	1	0.01	0.40-0.89
*Openness to experience*	−0.18	0.08	−0.44	0.83	−2.23	4.96	1	0.03	0. 71-0.98
*Perceived social support - family*	−0.07	0.04	−0.52	0.93	−1.95	3.78	1	0.05	0.87-1.00
*Perceived social support - significant others*	−0.07	0.04	−0.44	0.93	−1.91	3.65	1	0.06	0.87-1.00

*Overall model evaluation: Goodness-of-fit test: Hosmer & Lemeshow: χ^2^ = 35.07, df = 171, p < 0.01. Nagelkerke R^2^ = 0.265. Cox & Snell R^2^ = 0.178.*

*COVID19-Vaccine Uptake level ‘1’ coded as class 1. Variable coding: Marital Status 1 = in relation, 0 = single.*

*^+^Standardized estimates represent estimates where the continuous predictors are standardized (X-standardization).*

## Discussion

To our knowledge, this study represents the first attempt within the international literature to analyze COVID-19 vaccine uptake among family caregivers of people with dementia, and to identify some of the psychological and psychosocial characteristics relevant to COVID-19 vaccination behavior.

In our sample, we found a high percentage of COVID-19 vaccine uptake in both family caregivers (75.4%) and dementia patients (81.6%). Even these high numbers, however, leave about one quarter of caregivers unvaccinated. Only 48% of the participants believed that the vaccines would enable their family members to re-establish the social habits that they had before, such as leaving the home and attending dedicated health services. Because returning to normal is important, these findings suggest that increasing family caregivers’ confidence in the benefits of COVID-19 vaccines through specific information campaigns might be an effective strategy for moving some of the remaining unvaccinated caregivers into the vaccinated group.

Regarding sources of information, we found that family caregivers received vaccine information mainly from print or electronic newspapers (86.5%) and TV (81%) emphasizing that information campaigns focusing on increasing confidence in vaccine benefit should target delivery mostly in these two channels. The relatively high vaccine uptake observed in this sample may be due, in part, to the fact that information about the vaccines was not coming primarily from social media. Prior research has shown that negative views about vaccines are associated with obtaining health information online (e.g., Wilson and Wiysonge, 2020; Clark et al., 2022) where conspiracy theories abound, merge with other sources of misinformation, and are amplified with deleterious results (Stein et al., 2021). Stein and colleagues argue the need for a paradigmatic shift, away from misinformation- and conspiracy-related pseudo-environments; our data are consistent with this idea, and in addition to targeting the information sources that are most used, efforts should be made to highlight radio and online (especially social media) sources that are accurate and reliable.

Previous studies conducted with the general population provide somewhat conflicting results regarding associations between demographic variables and COVID-19 vaccine uptake. Our results are consistent with findings showing demographics to be unrelated to COVID-19 vaccine uptake (for a review see: AlShurman et al., 2021).

Regarding attitudes toward vaccination, we found that only worries about unforeseen future effects were significantly related to the COVID-19 vaccine uptake among family caregivers of people with dementia, indicating that family caregivers concerned about potential side effects of vaccines were less likely to have been vaccinated against COVID-19. This outcome may be partially explained by the rapid manner in which the COVID-19 vaccine was produced (Haynes, 2021) and suggest that these concerns should also be a direct focus of informational campaigns. Emphasizing the safety of vaccines, despite their rapid development and rollout, may be effectively accomplished both through public health campaigns and in the medical setting, with healthcare providers being encouraged to specifically address this issue when they communicate with patients.

Finally, in line with the study conducted in the Australian general population (Browne et al., 2015), we found that the personality trait “openness to experience” was negatively related to COVID-19 vaccine uptake among family caregivers of people with dementia. This means that family caregivers who were more imaginative, creative, inventive, open to unusual ideas, adventurous, and non-conforming (Salmon, 2012) were less likely to take the vaccine than those with lower levels of openness to experience. Interestingly, other findings suggest a positive relationship between openness to experience and the tendency to believe in conspiracy theories (Swami et al., 2010; Oortwijn, 2020), and it has been shown that a conspiracy mentality predicts vaccine hesitancy (Hornsey et al., 2018; Oortwijn, 2020). In future studies it could be relevant to investigate the possible mediating role of a conspiracy mentality in the relationship between openness to experience and vaccine uptake that we have found among family caregivers of people with dementia.

### The theoretical and practical contribution of the study

Considering that no studies have examined the choice of family caregivers of people with dementia to uptake COVID-19 vaccine, the present findings extend the knowledge base relative to this population. Specifically, the present study shows that greater worry about unforeseen future effects and higher openness to experience both predict lower COVID-19 vaccine uptake. In terms of the practical implications, our results suggest that targeting of the approximately one-fourth of caregivers who have opted not to be vaccinated should focus on safety of the vaccines, debunking conspiratorial myths, and emphasizing the wisdom a *choosing* a vaccine (vs. framing it as a conformist choice). Our findings also suggest that newspapers and television will be the best channels through which to deliver these interventions.

### Limitations of the study

The limitations of this research can provide helpful directives for future studies. First, it is essential to remember that convenience sampling (chosen due to time and financial constraints) is not random sampling. Second, the majority of family caregivers and patients were females. However, these data are in line with previous studies in the Italian population which show a higher female prevalence of both dementia (Bacigalupo et al., 2018) and family caregivers (Gagliardi et al., 2022). Future studies should be carried out in more gender balanced samples. Third, the response rate was not as high as would have been desired, although it was consistent with what is typically seen in voluntary, self-report studies with this sort of sampling approach. Future studies might utilize a more robust recruitment approach to increase the response rate. Fourth, the cross-sectional design adopted cannot reveal causality; although this is a limitation, it is not one that is likely to be rectified. Therefore, it serves merely as a caution, with regard to interpretation of results. Finally, self-reported measures were administered to assess the dimensions of this study. Although measures were carefully selected, the resultant survey was somewhat long, and the respondent burden (and consequent effects on responses) is unclear. Therefore, future research should consider different methods to reduce self-report biases and potential respondent fatigue.

## Conclusion

Overall, this study has some important strengths which may have bearing on clinical practice and future research, such as the identification of the links between attitudes toward vaccines (i.e., worries about unforeseen future effects), personality traits (i.e., openness to experience) and the choice to uptake COVID-19 vaccine among family caregivers of people with dementia. These data provide the initial bricks in an evidence-based foundation for setting up vaccination campaigns specifically targeting this group.

## Data Availability Statement

The raw data supporting the conclusions of this article will be made available by the authors, without undue reservation.

## Ethics statement

The studies involving human participants were reviewed and approved by Ethical Committee of Calabria Region (Catanzaro, Italy). The patients/participants provided their written informed consent to participate in this study.

## Author contributions

FB, AM, and LM wrote the manuscript. VL, FB, VI, CF, FG, ES, MF, RL, and FA collected data. FB, AM, and FC created database and tables. FC and AM performed statistical analysis. FB, RS, LM, RM, and AB conceived and designed the study. LM revised the language. All authors revised the manuscript and approved the submitted version.

## Conflict of Interest

The authors declare that the research was conducted in the absence of any commercial or financial relationships that could be construed as a potential conflict of interest.

## Publisher’s Note

All claims expressed in this article are solely those of the authors and do not necessarily represent those of their affiliated organizations, or those of the publisher, the editors and the reviewers. Any product that may be evaluated in this article, or claim that may be made by its manufacturer, is not guaranteed or endorsed by the publisher.
